# The Use of Miswak as Toothbrush for Orthodontic Patient

**DOI:** 10.1155/2016/7472340

**Published:** 2016-11-22

**Authors:** Khoirulzariah Ismail

**Affiliations:** Craniofacial and Biomaterial Sciences Cluster, Advanced Medical and Dental Institute, Universiti Sains Malaysia, Bertam, 13200 Kepala Batas, Pulau Pinang, Malaysia

## Abstract

This case report presents a patient who is undergoing orthodontic treatment with upper and lower fixed appliance. An interesting point on this case is that the patient only uses Miswak as her oral hygiene tool due to her religious belief. The oral hygiene protocol was allowed and her oral health was closely monitored throughout her orthodontic treatment.

## 1. Introduction

The Miswak is a stick made from the roots of the Arak tree (*Salvadora persica*). They are used as an oral hygiene tool mostly in the Middle Eastern and several African countries. Several studies have shown possible beneficial effect on the use of Miswak as a tooth brushing tool. The periodontal status among Sudanese Miswak users was shown to be better than normal toothbrush users [1]. Not only does the chewing stick remove plaque mechanically, it also has been reported to have positive antimicrobial activities that protect from cariogenic and periodontopathic bacteria when being used [2].

The use of Miswak among patients that are undergoing orthodontic treatment has not been well documented in the literature. A study among Arab orthodontic patients shows that only 8% of these patients use Miswak, but as an adjunct to their daily tooth brushing routine [3]. A fixed oral health care regiment has always been prescribed to patient to ensure optimum oral health level throughout orthodontic treatment. This case report would demonstrate the feasibility of using Miswak as part of oral hygiene care for orthodontic patient.

## 2. Case Presentation

A 19-year-old Malay girl medically fit and healthy attended Orthodontic Specialist Clinic, Advanced Medical & Dental Institute requesting for orthodontic treatment with the complaint of “front teeth sticking out.” Orthodontic assessment was performed and the patient had notified that she is using Miswak to clean her teeth and requested permission to continue her usual oral hygiene protocol whilst having her orthodontic treatment.

**Figure 1:**
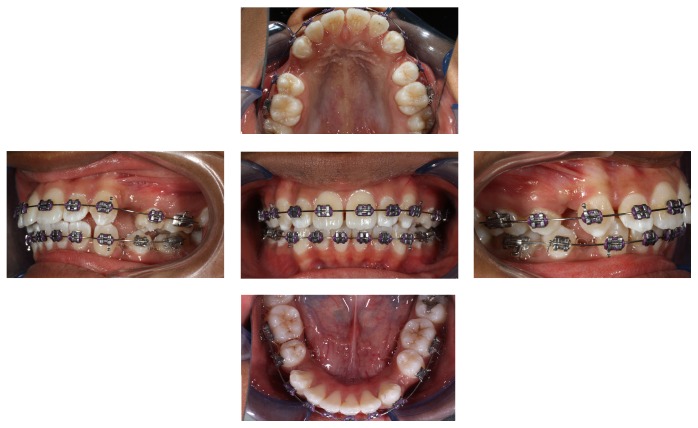
Intraoral pictures of patient, six months into fixed appliance treatment showing no detrimental changes to her oral health.

Since her oral hygiene and plaque score was good, I had agreed with her request. Risks of poor oral hygiene have been thoroughly discussed with patient when consent for orthodontic treatment was gained. Mid-treatment (Figure 1) clinical photographs show her oral hygiene status during orthodontic treatment. Her oral hygiene was in an optimal condition with minimal plaque. There were no signs of periodontal problem as well. The plaque score was assessed using Silness-Löe Plaque Index [4] on each of her visits. The plaque score was obtained by recording or grading the presence of plaque on specific tooth surfaces. By adding each of her tooth scores together and dividing it with the total number of teeth examined, the patient's Plaque Index is obtained. An index of less than 0.1 means no plaque. 0.1 to 1.0 indicates a small quantity of plaque, 1.1–2.0 a moderate amount, and between 2.1 and 3.0 a considerable one. Her plaque score pre- and mid-treatment can be assessed in Table 1. The patient is still undergoing her orthodontic treatment and is about to complete the alignment stage of treatment.

**Figure 2:**
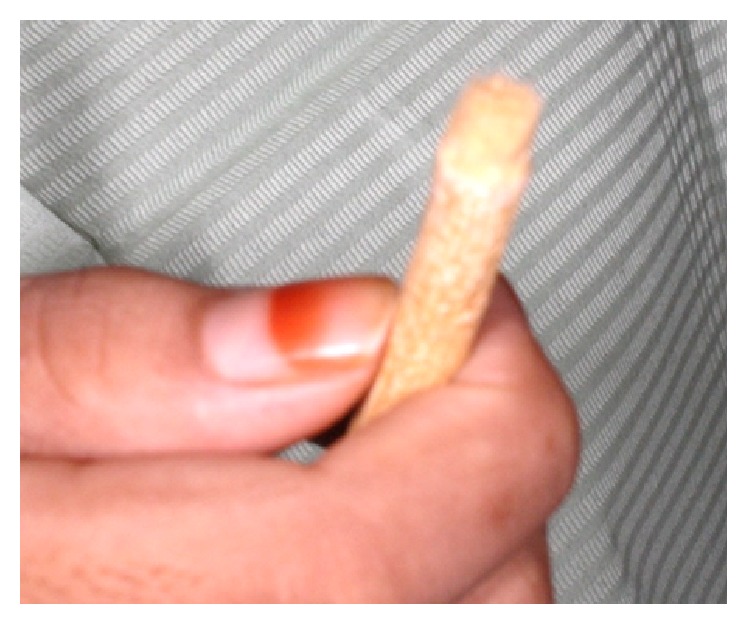
The Miswak plant use by the patient to clean her teeth. It works as a small tufted toothbrush to mechanically remove plaque from tooth surfaces.

**Figure 3:**
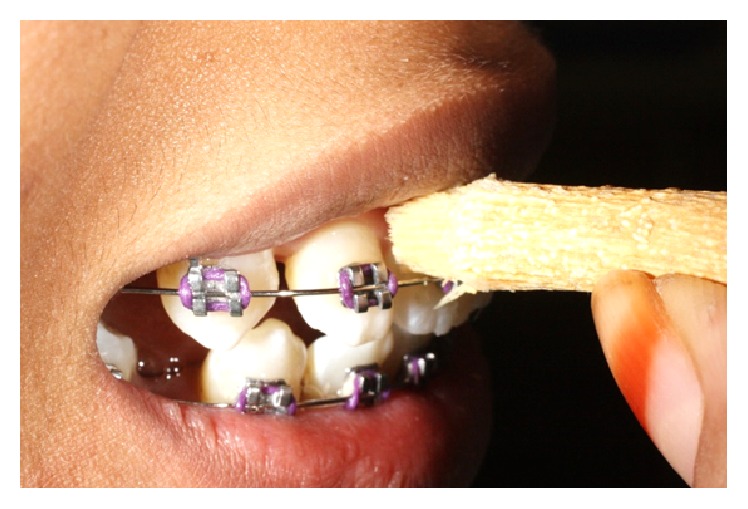
Patient demonstrated how she uses the Miswak plant.

The patient was asked to bring along her brushing tool to demonstrate how she uses it daily (Figure 2). The soft tufted end of the Miswak plant is almost similar to a small toothbrush. In Figure 3, patient demonstrates that she is able to maneuver the Miswak to clean in between her teeth and her brackets. She cleans the tooth surfaces in a circular or vibratory motion at one small surface and moves to the next adjacent surface.

## 3. Discussion

The use of Miswak as a tooth brushing tool is more common in Arabic country. Religious connotation is often associated with its use on daily hygiene practice as the use of Miswak was promoted by Prophet Muhammad [5]. A practice Muslim would try to follow the way of life of Prophet Muhammad, explaining the use of Miswak in non-Arab Muslim country such as Malaysia.

An interesting finding by al-Otaibi indicates that the use of Miswak was preferred by the less educated Saudi Arabians [6]. The use of toothbrush is the most common tool in oral hygiene care among Malaysian's Malays whilst the use of Miswak is evidently rare and has not been reported in the literature.

A clinical trial was performed in Jordan comparing different type of oral hygiene tool among orthodontic patients with fixed appliance. The study found that the combined use of Miswak and toothbrush gave the best plaque control result [7]. Interestingly, this patient claimed that she did not use any other oral health care tool (toothpaste, mouthwash, toothbrush, interdental toothbrush, or floss) other than the Miswak plant. This might indicate the patient has a good mechanical plaque removal technique when using the Miswak.

Presence of fixed appliance intraorally is considered as a leading factor that causes hindrance for patients to properly clean their teeth [8]. This would cause a multitude of side effects mainly, periodontal problem. A study performed to assess factors that cause white spot lesion in patients with fixed appliance has concluded that poor oral hygiene was the highest risk that contributes to the development of enamel decalcification [9].

Oral hygiene condition of an orthodontic patient is a great concern among clinician. Ideally, an orthodontic treatment should be carried out in patient that has an optimal oral hygiene. Routine screening for oral hygiene is always performed by an orthodontist prior to embarking treatment. This would aid clinician in giving proper care to patients and prevent the occurrence of adverse side effect during treatment.

## 4. Conclusion

From this case, it can be concluded that a fixed regiment of oral hygiene should not be prescribed to all patients. The hygiene protocol should be given on a case by case basis depending on patients' oral hygiene status and their physical and mental capabilities in taking care of their oral health. The use of Miswak can be considered as an option in oral hygiene management of orthodontic patient.

## Figures and Tables

**Table 1:** Plaque score of patient using Silness-Löe Plaque Index.

	Pretreatment (prior to bond-up)	3 months' mid-treatment	6 months' mid-treatment
Plaque Index	1.0	1.2	1.0
